# Accuracy of Repetitive Ocular Vestibular–Evoked Myogenic Potentials to Diagnose Myasthenia Gravis in Patients With Ptosis or Diplopia

**DOI:** 10.1212/WNL.0000000000209395

**Published:** 2024-04-26

**Authors:** Yulia Valko, Magdalena A. Wirth, Fabienne C. Fierz, Marianne K. Schesny, Sally Rosengren, Tanja Schmückle-Meier, Christopher J. Bockisch, Dominik Straumann, Bettina Schreiner, Konrad P. Weber

**Affiliations:** From the Neurology Department (Y.V., M.K.S., C.J.B., D.S., B.S., K.P.W.), Clinical Neuroscience Center, and Ophthalmology Department (M.A.W., F.C.F., T.S.-M., C.J.B., K.P.W.), University Hospital Zurich, University of Zurich, Switzerland; Neurology Department and Institute of Clinical Neurosciences (S.R.), Royal Prince Alfred Hospital, Camperdown; and Central Clinical School (S.R.), Faculty of Medicine and Health, University of Sydney, Australia.

## Abstract

**Background and Objectives:**

We developed repetitive ocular vestibular–evoked myogenic potentials (roVEMP) as an electrophysiologic test that allows us to elicit the characteristic decrement of extraocular muscles in patients with ocular myasthenia gravis (OMG). Case-control studies demonstrated that roVEMP reliably differentiates patients with OMG from healthy controls. We now aimed to evaluate the diagnostic accuracy of roVEMP for OMG diagnosis in patients with ptosis and/or diplopia.

**Methods:**

In this blinded prospective diagnostic accuracy trial, we compared roVEMP in 89 consecutive patients presenting with ptosis and/or diplopia suspicious of OMG with a multimodal diagnostic approach, including clinical examination, antibodies, edrophonium testing, repetitive nerve stimulation of accessory and facial nerves, and single-fiber EMG (SFEMG). We calculated the roVEMP decrement as the ratio between the mean of the first 2 responses compared with the mean of the sixth-ninth responses in the train and used cutoff of >9% (unilateral decrement) in a 30 Hz stimulation paradigm.

**Results:**

Following a complete diagnostic work-up, 39 patients (44%) were diagnosed with ocular MG, while 50 patients (56%) had various other neuro-ophthalmologic conditions, but not MG (non-MG). roVEMP yielded 88.2% sensitivity, 30.2% specificity, 50% positive predictive value (PPV), and 76.5% negative predictive value (NPV). For comparison, SFEMG resulted in 75% sensitivity, 56% specificity, 55.1% PPV, and 75.7% NPV. All other diagnostic tests (except for the ice pack test) also yielded significantly higher positive results in patients with MG compared with non-MG.

**Discussion:**

The study revealed a high sensitivity of 88.2% for roVEMP in OMG, but specificity and PPV were too low to allow for the OMG diagnosis as a single test. Thus, differentiating ocular MG from other neuro-ophthalmologic conditions remains challenging, and the highest diagnostic accuracy is still obtained by a multimodal approach. In this study, roVEMP can complement the diagnostic armamentarium for the diagnosis of MG.

**Classification of Evidence:**

This study provides Class I evidence that in patients with diplopia and ptosis, roVEMP alone does not accurately distinguish MG from non-MG disorders.

**Trial Registration Information:**

ClinicalTrials.gov: NCT03049956.

## Introduction

In our previous work, we showed that repetitive ocular vestibular–evoked myogenic potentials (roVEMP) allow us to directly detect the characteristic muscle decrement in the affected extraocular muscles of patients with ocular myasthenia gravis (MG).^1^ This proof-of-concept study with a case-control design demonstrated that roVEMP reliably differentiated between MG patients with ocular involvement and healthy control participants. A follow-up study confirmed these findings and suggested a repetition rate of 30 Hz as the optimal stimulation parameter for eliciting an extraocular muscle response decrement.^2^ Another case-control study provided Class III evidence that roVEMP distinguishes MG from other neuromuscular diseases with ocular symptoms.^3^

The traditional ancillary tests, including assays for autoantibodies directed against components of the neuromuscular endplate, edrophonium testing, repetitive nerve stimulation (RNS), and single-fiber EMG (SFEMG), demonstrate a lower sensitivity in isolated ocular compared with generalized MG.^4^ Particularly promising, thus, was the fact that roVEMP turned out to be the only diagnostic procedure with similarly high diagnostic accuracy in both isolated ocular and generalized MG patients, whereas all other tests were less sensitive in patients with isolated ocular MG.^1^

To establish roVEMP as a helpful test for ocular MG in clinical practice, we now set out to validate roVEMP in a blinded prospective diagnostic accuracy study in a neuro-ophthalmologic cohort consisting of consecutive patients presenting with diplopia and/or ptosis. We aimed to compare the diagnostic accuracy of roVEMP with established neurophysiologic tests, including SFEMG. The primary research question was to determine the diagnostic accuracy of roVEMP to distinguish patients with ocular MG from patients without MG but with ptosis and/or diplopia (non-MG).

## Methods

### Standard Protocol Approvals, Registrations, and Patient Consents

This study was conducted at the Interdisciplinary Center for Vertigo and Neurological Visual Disorders, University Hospital Zurich, Switzerland. It has been designed according to the tool for Quality Assessment of Diagnostic Accuracy Studies.^5^ All participants provided written informed consent in accordance with the Declaration of Helsinki. The study was approved by the local ethics committee (Kantonale Ethik-Kommission Zurich, BASEC-No. 2016-01109) and is registered under ClinicalTrials.gov (NCT03049956).

### Study Participants

Ninety-six consecutive patients 18 years or older with diplopia and/or ptosis suspicious for MG were included in this prospective study. They were recruited over 3 years, from November 2016 to December 2019, and gave their written informed consent. We did not exclude patients with additional generalized symptoms because these sometimes became apparent only during the diagnostic process in the study. We excluded patients with known vestibular disorders from the study because vestibular dysfunction may interfere with oVEMP testing. Moreover, we did not include pregnant patients (a pregnancy test was performed in every female patient of childbearing age). Seven patients did not complete the study because they either withdrew their participation or were lost to follow-up.

### Diagnostic Procedures

All patients underwent a standardized clinical assessment including careful history and physical examination. The core of the clinical assessment was the rating of disease severity with the Besinger score, a well-established clinical score for patients with MG.^6^ All patients underwent antibody testing, including autoantibodies against acetylcholine receptor (AChR-Ab), titin, muscle-specific kinase, and low-density lipoprotein receptor-related protein 4. Clinical and pharmacologic diagnostic procedures comprised the Simpson test with occurrence or increase of ptosis while looking upward^7^ within 60 seconds, the ice pack test with improvement of ptosis with local cooling for 2 minutes,^8^ and a video-documented edrophonium test.^9^ In the edrophonium test, we first injected 2 mg edrophonium and verified that no acute adverse reactions occurred; if this was not the case after 30 seconds, the remaining 8 mg edrophonium were injected (total 10 mg). The test was performed on a recliner with a pulse oximeter and atropine ready. Objective improvement of ptosis and/or diplopia was assessed, and all tests were documented on video. Contraindications for the test were asthma or cardiac disease. In addition to roVEMP, we performed other electrophysiologic tests, including stimulated SFEMG of orbicularis oculi muscles and 3 Hz RNS of both facial nerves (nasalis recording) and 1 accessory nerve (trapezius recording) at rest, twice for each muscle with an interval of 2 minutes between the stimulations, where the decrement between the first and fifth response of ≥10% was defined as abnormal.^10^ Patients were withdrawn from acetylcholine-esterase inhibitors for at least 10 hours when the roVEMP and electrophysiologic testing were performed after initiation of treatment.

The final diagnosis (MG yes/no) was established at a 3-month follow-up visit based on all the clinical tests and evaluation of any treatment response. Owing to the SARS-CoV-2 pandemic, some patients were unable to visit the site and were followed up by phone. The study physicians who made the final diagnosis were blinded to the roVEMP results but had access to all the clinical routine tests. Conversely, both the examiners (M.A.W., Y.V., F.C.F.) and the analyzer (Y.V.) of roVEMP were blinded to the final clinical diagnosis. MG severity was classified according to the Myasthenia Gravis Foundation of America (MGFA) classification.^11^

### Repetitive Ocular Vestibular–Evoked Myogenic Potentials

We have used the same setup for roVEMP measures as described earlier.^1,2^ We examined patients while they were in the supine position with their heads supported by a pillow. For optimal recording quality, we asked the patients to look up for each trial and rest with their eyes closed in between.^12,13^ Using a hand-held “minishaker” (model 4810; amplifier model 2706, Brüel & Kjaer A/S) positioned over the hairline near Fz, we delivered repetitive bone-conducted 500 Hz vibration bursts of 4 milliseconds at 30 Hz.^14^ To minimize electromagnetic interference, the minishaker was shielded with a custom-built μ-metal encasement. We recorded roVEMPs binocularly using active electrodes (Blue sensor N, Ambu A/S) placed just below the lower eyelid, and reference electrodes placed directly below on the cheek, while the earth was placed on the chin.^15^ We used a laboratory data acquisition system to produce the experimental stimuli and to record the surface EMG signals (power 1401 and 1902 preamplifier; Cambridge Electronic Design CED, Cambridge, United Kingdom). The signals were bandpass filtered (5 Hz–2,000 Hz) and sampled at 10 kHz using a sweep-based recording software (Signal, version 5; Cambridge Electronic Design CED).

We calculated the roVEMP decrement as the ratio (percentage) between the mean of the first 2 responses compared with the mean of the sixth-ninth responses in the train. To test the diagnostic accuracy of roVEMP, we used a cutoff of >9% (unilateral decrement) in a 30 Hz stimulation paradigm, as prespecified by a previous study.^2^ A unilateral decrement implies that at least one of the 2 eyes showed a decrement and refers to the eye with the larger decrement. The roVEMP was conducted in a separate session by independent examiners (M.A.W., Y.V. or F.C.F.).

### Single-Fiber EMG

Eighty-six patients underwent stimulated SFEMG of orbicularis oculi muscles with disposable SFEMG needles (25 × 0.45 mm; SEI EMG s.r.l., Citadella, Italy) using the SFEMG module of a VikingSelect EMG system (Natus Medical Inc., Pleasanton, CA). The same independent examiners (K.P.W., B.S.) recorded all SFEMG together. The analysis was based on our own normal SFEMG values for jitter from 13 healthy participants, where 1 participant had to be excluded because of noisy signals. The mean age of the 12 included normal participants was 36 ± 9 years with 6 male and 6 female persons. We calculated the abnormality of the jitter according to a previous study^16^ and investigated 23 ± 2 fiber pairs for each participant. The upper limit (cutoff) of the mean consecutive difference (MCD) value was defined as the mean value of all MCD +2 SD and was 36 μs in our normal participants. Outliers were defined as MCD upper limit +2 SD from all individual jitters and were >47 μs, where the detection of ≥3 outliers was defined as abnormal.^16^ Increased jitter was defined as mean MCD >36 μs or detection of ≥3 outliers.

### Data Processing and Statistics

The roVEMP data were analyzed with custom software written in MatLab (The MathWorks Inc., Natick, MA). Outlier trials were removed with a median absolute deviation algorithm,^17^ complemented by visual inspection. Twelve patients with insufficient roVEMP data were excluded. We report the results as recommended by the Standards for Reporting of Diagnostic Accuracy Studies (STARD)^18^ to assure the requirements for later Cochrane diagnostic accuracy reviews^19^ (STARD flowchart). We expressed the diagnostic accuracy of the various diagnostic tests by calculating sensitivity and specificity as well as positive and negative predictive values (PPV, NPV), and used 95% confidence intervals (95% CI) to quantify statistical uncertainty. Based on these parameters, we additionally calculated the resulting accuracy for roVEMP and all other diagnostic tests.

We used IBM SPSS (Chicago, IL) version 26.0 for statistical analysis. Group data were described by means and SDs, unless otherwise specified. For comparison of normally distributed data, we applied unpaired Student *t* tests. χ^2^ tests were used for nominal data. For nonparametric data, we applied the Mann-Whitney *U* test. Significance was accepted at *p* < 0.05.

### Sample Size Calculation

To estimate the number of patients with and without MG needed for a successful outcome of the study, sample size was calculated with MedCalc software version 15.6 (Ostend, Belgium). Sample size calculation was based on the area under the receiver operator characteristic curve^20,21^ of our roVEMP stimulation test (area under the curve = 0.78), as determined in our proof-of-concept study.^1^ It was estimated that one in 5 patients meeting the entry criteria for the study would be ultimately diagnosed with MG, as confirmed by the reference standard. According to the sample size calculation, a minimum of approximately 60 participants had to be recruited to reliably determine the diagnostic accuracy of the test. We used the McNemar test to determine whether the sensitivities of the roVEMP and SFEMG were statistically different or not.

### Data Availability

Anonymized data not published within this article will be made available by request from any qualified investigator.

## Results

### Participants

The main demographic and clinical characteristics of the 39 patients classified as MG and the 50 patients classified as non-MG are summarized in Table 1. MG and non-MG patients did not differ about age (63 ± 16 years vs 60 ± 16 years, *p* = 0.33), but female sex was less common in MG (41%) than in non-MG patients (64%). The clinical presentation did not differ between MG and non-MG patients, including similar prevalence of diplopia (72% vs 68%) and ptosis (79% vs 76%) as well as similar Besinger scores (5.1 ± 3.1 vs 4.1 ± 2.7). Symptom duration, however, was shorter in MG patients (9 ± 19 months) than in non-MG patients (29 ± 31 months) (*p* = 0.001).

**Table 1:** Demographic and Clinical Characteristics in 39 Patients Classified as MG and 50 Patients With Various Other Neuro-Ophthalmologic Diagnoses (Non-MG)

	Non-MG patients (n = 50)	MG patients (n = 39)	*p* Value
Age, y	60 ± 16	63 ± 16	0.33
Female sex, n	32 (64%)	16 (41%)	0.04
Duration of symptoms, mo	29 ± 31	9 ± 19	0.001
Besinger score	4.1 ± 2.7	5.1 ± 3.1	0.10
Diplopia	34 (68%)	28 (72%)	0.82
Ptosis	38 (76%)	31 (79%)	0.80
Thymoma	0	4	
Early onset MG (<50 y old at onset)		8	

Abbreviation: MG = myasthenia gravis.

MG severity according to the MGFA classification was I in 25 MG patients, IIa in 7 MG patients, and IIb in 7 MG patients. Five MG patients (13%) were thymoma-associated and 10 MG patients (26%) with early-onset.

Alternative diagnoses in non-MG patients included decompensated exophoria resp. esophoria (n = 10), mitochondrial myopathy (n = 6), transitory ischemic attack (n = 4), isolated weakness of m. levator palpebrae (n = 3), Miller-Fisher syndrome (n = 3), constitutional ptosis (n = 3), Horner syndrome (n = 2), hordeolum resp. unclear local process in eyelid (n = 2), multiple cranial neuropathy of unknown cause (n = 2), oculopharyngeal muscular dystrophy (n = 2), levator aponeurosis dehiscence (n = 2), oculomotor nerve palsy (n = 1), ptosis secondary to contact lens use (n = 1), symptoms due to orbital fracture (n = 1), age-related ptosis (n = 1), endocrine orbitopathy (n = 1), and eyelid apraxia due to multiple sclerosis (n = 1) with many patients having multiple possible diagnoses. In 6 patients, the diagnosis remained unclear.

No adverse events occurred from performing roVEMP or other ancillary tests.

### Diagnostic Test Findings

**Figure 1:**
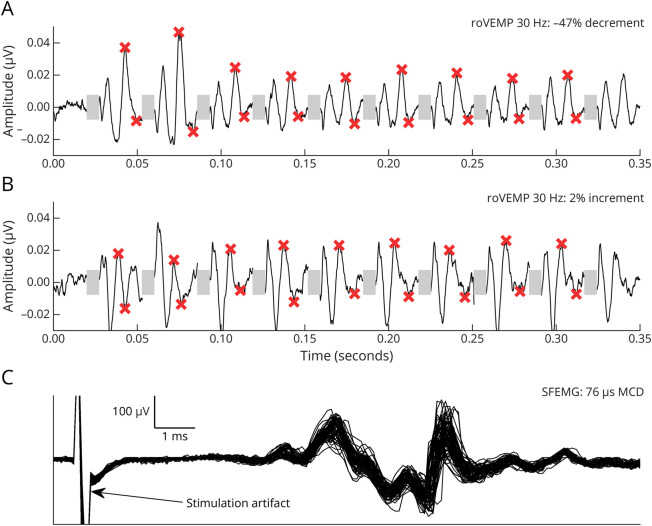
Single-Subject Data of a Subject With (A) and Without (B) Pathologic Decrement at 30 Hz Stimulus Repetition Rates The gray bars indicate the 10 stimuli at 30 Hz repetition rates. The magnitude of each oVEMP response represents the sum of the second peak and second trough (asterisks). Decrement is defined as the ratio between the mean of the first 2 responses compared with the mean of the sixth-ninth responses. Single-subject data of a patient with increased jitter in SFEMG (C). roVEMP = repetitive ocular vestibular–evoked myogenic potentials; SFEMG = single-fiber EMG.

**Figure 2:**
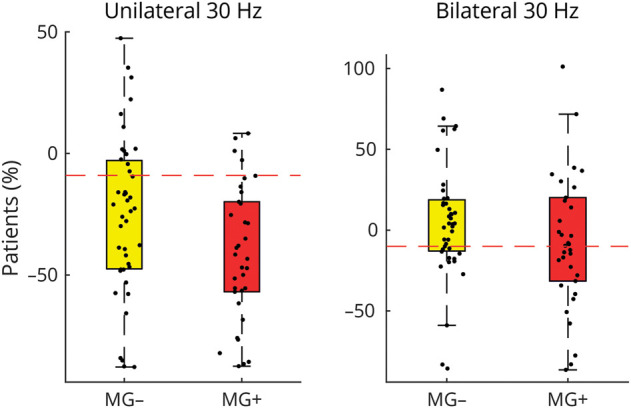
Group Comparison of Unilateral and Bilateral Decrements Between Patients With MG (MG+) and Without MG (MG−) The dashed red lines indicate the predefined diagnostic thresholds for unilateral (i.e., eyes with the larger decrement) and bilateral decrement (i.e., eyes with the smaller decrement) using 30 Hz roVEMP stimulations. The boxes demonstrate first and third quartiles, while the ends of the whiskers represent the most extreme data points without outliers. MG = myasthenia gravis; roVEMP = repetitive ocular vestibular–evoked myogenic potentials.

Figure 1 demonstrates typical roVEMP results at 30 Hz with (A) and without (B) decrement as well as SFEMG with increased jitter (C). We found significant group differences in roVEMP measures for mean unilateral decrements (using the eye with the larger decrement) (Table 2; Figure 2). Age, sex, disease duration, early vs late onset, presence/absence of thymoma, and ocular symptoms—that is, only diplopia, only ptosis or both diplopia/ptosis—had no effect on the magnitude of decrements.

**Table 2:** Group Comparison Between the Diagnostic Tests in MG Patients and in Neuro-Ophthalmologic Patients Without MG (Non-MG)

	Non-MG patients (n = 50)	MG patients (n = 39)	*p* Value
roVEMP (30 Hz stimulation)			
N	43	34	
Unilateral decrement	−25.1 ± 32.7	−41.2 ± 27.8	0.02
Positive result (cutoff −9%)	30/43 (70%)	30/34 (88%)	0.046
Single-fiber EMG			
SFEMG, pos. result (n)	22/50 (44%)	27/36 (76%)	0.004
Mean MCD	31.6 ± 16.7	50.4 ± 27.9	<0.001
Increased jitter	3.5 ± 4.3	8.4 ± 6.5	<0.001
Blocked impulse	0.4 ± 0.9	2.1 ± 2.2	<0.001
RNS (3 Hz)			
Accessory nerve	1/48 (2%)	13/36 (36%)	<0.001
Facial nerve	4/48 (8%)	16/36 (44%)	<0.001
Positive RNS (accessory and/or facial nerves)	5/48 (10%)	20/36 (56%)	<0.001
Clinical and antibody testing			
Simpson test, pos. (n)	14/46 (30%)	25/38 (66%)	0.003
Ice pack test, pos. (n)	9/30 (30%)	13/21 (62%)	0.07
Edrophonium test, pos. (n)	4/40 (10%)	29/31 (94%)	<0.001
Anti-AChR, pos. (n)	2/50 (4%)	33/39 (85%)	<0.001
Anti-titin, pos. (n)	2/50 (4%)	19/39 (49%)	<0.001
Anti-MuSK, pos. (n)	0/50 (0%)	1/39 (2.5%)	0.44
Anti-LRP4, pos. (n)	0/50 (0%)	2/39 (5%)	0.19

Abbreviations: AChR = acetylcholine receptor; LRP4 = lipoprotein receptor-related protein 4; MCD = mean consecutive difference; MG = myasthenia gravis; MuSK = muscle-specific kinase; RNS = repetitive nerve stimulation; roVEMP = repetitive ocular vestibular–evoked myogenic potentials.

**Figure 3:**
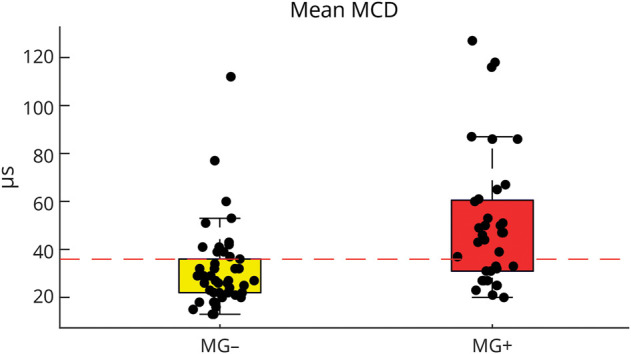
MCD Values in Single-Fiber EMG Were Significantly Higher in Patients With MG (MG+) Than in Non-MG (MG−) The dashed red line indicates the cutoff (>36 μs) for increased jitter as defined in our neuromuscular unit. The boxes demonstrate first and third quartiles, while the ends of the whiskers represent the most extreme data points without outliers. MCD = mean consecutive difference; MG = myasthenia gravis.

Except for the ice pack test, all diagnostic procedures exhibited significantly higher positive test results in MG patients compared with non-MG patients (Table 2), including significantly higher mean MCD values in SFEMG (Figure 3).

### Diagnostic Accuracies

Table 3 summarizes the diagnostic accuracy measures of roVEMP, SFEMG, and the other ancillary tests. The detection rate for MG by roVEMP was very sensitive (88.2%), but not specific (30.2%); PPV/NPV was 50.0/76.5%. Compared with roVEMP, SFEMG seemed to have a lower sensitivity (75%) and a higher specificity (56%), but these differences were not statistically significant (McNemar; *p* = 0.18 and *p* = 0.58); PPV/NPV was 55.1/75.7%. A positive edrophonium test (94%) and AChR-antibody positivity (85%) identified MG patients with the highest sensitivity while also yielding relatively high specificities/PPV/NPV of 90%/87.9%/94.7% and 96%/94.3%/88.9%, respectively. Similar to the diagnostic accuracy findings in roVEMP and SFEMG, and despite the significant group differences, most of the other diagnostic procedures had either a low sensitivity (51% in RNS of accessory and/or facial nerves) or a low specificity (56%–70% in SFEMG, and Simpson test).

**Table 3:** Overview on Diagnostic Accuracy, Including Sensitivity, Specificity, PPV, NPV, and Accuracy Expressed as Percentages, as Well as Positive Likelihood Ratios, With 95% CI

Diagnostic test	Sensitivity, % (95% CI)	Specificity, % (95% CI)	PPV, % (95% CI)	NPV, % (95% CI)	Accuracy, % (95% CI)	Positive likelihood ratio (95% CI)
roVEMP						
30 Hz unilateral decrement (cutoff −9% decrement)	88.2 (72.6–96.7)	30.2 (17.2–46.1)	50.0 (44.2–55.8)	76.5 (53.8–90.1)	55.8 (44.1–67.2)	1.3 (1.0–1.6)
Positive RNS (accessory and/or facial nerves)	51.3 (34.8–67.6)	90.0 (78.2–96.7)	80.0 (62.6–81.9)	70.3 (59.3–93.2)	73.0 (59.1–81.5)	5.1 (2.1–12.4)
Single-fiber EMG	75.0 (57.8–87.9)	56.0 (41.3–70.0)	55.1 (46.0–63.9)	75.7 (62.7–85.2)	64.0 (52.9–74.0)	1.7 (1.2–2.5)
Clinical and antibody testing						
Simpson test	65.8 (48.7–80.4)	69.6 (54.3–82.3)	64.1 (52.2–74.5)	71.1 (60.4–79.9)	67.9 (56.8–77.6)	2.2 (1.3–3.5)
Ice pack test	61.9 (38.4–81.9)	70.0 (50.6–85.3)	59.1 (43.2–73.3)	72.4 (59.2–82.6)	66.7 (52.1–79.2)	2.1 (1.1–3.9)
Edrophonium test	93.6 (78.6–99.2)	90.0 (76.3–97.2)	87.9 (74.0–94.9)	94.7 (82.4–98.6)	91.6 (82.5–96.8)	9.4 (3.7–23.8)
Anti-AChR antibodies	84.6 (69.5–94.1)	96.0 (86.3–99.5)	94.3 (80.8–98.5)	88.9 (79.3–94.4)	91.0 (83.1–96.0)	21.2 (5.4–82.8)
Anti-titin antibodies	48.7 (32.4–65.2)	96.0 (86.3–99.5)	90.5 (70.2–97.5)	70.6 (63.8–76.6)	75.3 (65.0–83.8)	12.2 (3.0–49.2)

Abbreviations: AChR = acetylcholine receptor; MG = myasthenia gravis; NPV = negative predictive value; PPV = positive predictive value; RNS = repetitive nerve stimulation; roVEMP = repetitive ocular vestibular–evoked myogenic potentials.

### Classification of Evidence

This study provides Class I evidence that in patients with diplopia and ptosis, roVEMP alone does not accurately distinguish MG from non-MG disorders.

## Discussion

Although our previous studies provided Class III evidence of roVEMP's ability to distinguish patients with ocular MG from healthy controls in a case-control design,^1,2^ this study aimed at validating the diagnostic utility of roVEMP for the diagnosis of ocular MG in clinical practice. In a carefully designed blinded diagnostic accuracy study with a before-after design, we included 89 consecutive patients presenting with ptosis and/or diplopia at our neuro-ophthalmology clinic and performed roVEMP, and all the established ancillary tests to confirm or refute the initial suspicion of ocular MG. We found that roVEMP at 30 Hz stimulation rates demonstrated significantly larger mean unilateral decrements in MG patients compared with non-MG patients. Our predefined cutoff of a >9% unilateral decrement revealed a high sensitivity of 88.2% but a low specificity of 30.2%, resulting in a positive predictive value of 50.0% and a negative predictive value of 76.5%. Accordingly, roVEMP had a moderate diagnostic accuracy of 55.8% for the identification of ocular MG. Considering the high sensitivity yet low specificity, the strength of roVEMP lies mainly in ruling out MG, that is, roVEMP is a so-called “SnNOut” test (sensitive, negative, out).^22^

Overall, the magnitude of decrements obtained at 30 Hz stimulation rates was comparable with our previous studies,^1,2^ which was expected as we used the same technical setup and practice parameters. roVEMP rarely produced significant decrements in extraocular muscles of healthy controls, but this study indicates that decrements are not specific to ocular MG, but may also occur in other neuro-ophthalmologic conditions, not due to MG.

Similarly, RNS has been reported to produce decremental responses in skeletal muscles affected by neuromuscular disorders other than MG, including amyotrophic lateral sclerosis.^23^ Nevertheless, RNS-induced decrements usually exhibit a high specificity for MG of 70%–100%, even when using patients with other neuromuscular disorders as positive controls.^24-27^ These studies, however, have been performed in patients with generalized MG, not ocular MG.

By comparison, SFEMG, which is currently considered the gold standard for electrophysiologic diagnosis of MG, also demonstrated only moderate diagnostic accuracy (sensitivity 75%, specificity 56%, PPV 55.1%, NPV 75.7%) in our hands, although we used a standardized stimulated SFEMG recording protocol with single-fiber needles.^16,28,29^ Several factors may affect the diagnostic accuracy of SFEMG, as suggested by highly variable sensitivity (64%–100%) and specificity (22%–100%) values reported in the literature.^27^ These differences probably arise from the high expertise required for performing the test, which is only available in highly specialized centers. For this reason, we minimized any possible examiner bias by using a stimulated SFEMG protocol and having all SFEMG examinations performed by the same experienced physicians. A possible limitation, however, is that the age of our included patients was considerably higher than that of our normative SFEMG laboratory cohort.

The test results of the other clinical bedside tests revealed a similar picture. Although the ice pack test failed producing a statistically significant difference (*p* = 0.07) in positive test results between ocular MG and non-MG, all other tests showed significantly higher positivity rates in ocular MG. As expected, edrophonium and anti-AChR antibody testing yielded the highest diagnostic accuracy (sensitivity, specificity, PPV, and NPV all >80%), but specificity was >90% also for anti-titin antibody testing and for RNS of accessory and/or facial nerves. The diagnostic accuracies of the latter were, however, restricted by low sensitivities of approximately 50%.

The relatively high number and magnitude of decremental responses elicited by roVEMP in our non-MG group challenge the specificity and, thus, the clinical utility of roVEMP as a sole diagnostic test in ocular MG. Several reasons may account for the low specificity. First, the physiologic requirements of extraocular muscles for quick and precise eye movements strongly differ from skeletal muscles, which are reflected by unique histologic and physiologic features, including very small motor units and twitch fibers with particularly high firing rates.^30-32^ One consequence is a high susceptibility of extraocular muscles in MG, explaining why ocular symptoms often appear first at disease onset. Hence, the question arises whether a decremental roVEMP response necessarily implies a defective neuromuscular transmission or whether other mechanisms may be responsible for fatigability and rapid exhaustion of extraocular muscles in non-MG patients. Second, roVEMP is anatomically based on multiple structures between the inner ear and extraocular muscles, making confounding factors more likely to occur.

In addition, several demographic, clinical, and technical variables may influence oVEMP results, including vestibular comorbidity, electrode montage, and gaze elevation during testing.^12,33,34^ However, since our MG and non-MG patients were well age-matched, vestibular dysfunction represented an exclusion criterion, and the roVEMP examination was performed in a very systematic and blinded fashion, we do not believe that one of these confounders can be blamed for having produced so many false positive roVEMP results. We must acknowledge, however, that MG and non-MG patients were not matched about sex, with significantly more men among the MG group. This higher prevalence of male patients with MG is due to the older age of included patients and reflects the well-known sex distribution among MG, with women having more often an earlier disease onset and male patients with MG having more often a disease onset at an older age. Although men have been reported to have higher oVEMP amplitudes than women,^35,36^ we did not observe any sex-related differences in the magnitude of decrements among our patients.

Theoretically, a high number of clinically missed MG diagnoses among patients eventually assigned to the non-MG group might have contributed to the low specificity of roVEMP. Considering the systematic and comprehensive diagnostic work-up, which was in line with the current diagnostic accuracy guidelines^10^ and included the response to treatment at a 3-month follow-up, this scenario seems unlikely. More likely, the diagnostically heterogeneous group of non-MG patients, including nonneurological problems (e.g., aponeurotic ptosis), and the varied disease duration in both groups also contributed to the relatively low roVEMP specificity.

Overall, the diagnostic accuracy variables of the other clinical bedside tests were comparable with the values published in the literature over the past 20 years,^27^ with the exceptions of the ice test, which had no diagnostic value and a remarkably high anti-AChR seropositivity (85%). The high proportion of patients with seropositive MG may be due to 14 patients were ultimately classified not as purely ocular but as generalized MG MGFA class II.

Future studies need to investigate how the current roVEMP practice parameters can be further optimized to achieve improved diagnostic accuracy, not only in studies with a case-control design but also in clinical practice so that roVEMP finds its place as a useful tool for the work-up of patients with MG.

In conclusion, ocular MG remains a challenging diagnosis that cannot be established with a single test. All the different tests have their own advantages and limitations, including different properties in sensitivity and specificity, but also in availability (e.g., edrophonium test) or dependence on expertise (e.g., SFEMG and roVEMP). It remains, thus, necessary to optimize the diagnostic yield by an appropriate combination of all available diagnostic tests.

## Appendix Authors

**Table:**

Name	Location	Contribution
Yulia Valko, MD	Neurology Department, Clinical Neuroscience Center, University Hospital Zurich, University of Zurich, Switzerland	Drafting/revision of the manuscript for content, including medical writing for content; major role in the acquisition of data; study concept or design; analysis or interpretation of data
Magdalena A. Wirth, MD	Ophthalmology Department, University Hospital Zurich, University of Zurich, Switzerland	Drafting/revision of the manuscript for content, including medical writing for content
Fabienne C. Fierz, MD	Ophthalmology Department, University Hospital Zurich, University of Zurich, Switzerland	Drafting/revision of the manuscript for content, including medical writing for content
Marianne K. Schesny, MD	Neurology Department, Clinical Neuroscience Center, University Hospital Zurich, University of Zurich, Switzerland	Drafting/revision of the manuscript for content, including medical writing for content
Sally Rosengren, PhD	Neurology Department and Institute of Clinical Neurosciences, Royal Prince Alfred Hospital, Camperdown; Central Clinical School, Faculty of Medicine and Health, University of Sydney, Australia	Drafting/revision of the manuscript for content, including medical writing for content; study concept or design
Tanja Schmückle-Meier	Ophthalmology Department, University Hospital Zurich, University of Zurich, Switzerland	Drafting/revision of the manuscript for content, including medical writing for content
Christopher J. Bockisch, PhD	Neurology Department, Clinical Neuroscience Center, and Ophthalmology Department, University Hospital Zurich, University of Zurich, Switzerland	Drafting/revision of the manuscript for content, including medical writing for content; analysis or interpretation of data
Dominik Straumann, MD	Neurology Department, Clinical Neuroscience Center, University Hospital Zurich, University of Zurich, Switzerland	Drafting/revision of the manuscript for content, including medical writing for content; study concept or design
Bettina Schreiner, MD	Neurology Department, Clinical Neuroscience Center, University Hospital Zurich, University of Zurich, Switzerland	Drafting/revision of the manuscript for content, including medical writing for content; analysis or interpretation of data
Konrad P. Weber, MD	Neurology Department, Clinical Neuroscience Center, and Ophthalmology Department, University Hospital Zurich, University of Zurich, Switzerland	Drafting/revision of the manuscript for content, including medical writing for content; study concept or design; analysis or interpretation of data

## Glossary

AChR: acetylcholine receptor
MCD: mean consecutive difference
MG: myasthenia gravis
MGFA: Myasthenia Gravis Foundation of America
NPV: negative predictive value
PPV: positive predictive value
RNS: repetitive nerve stimulation
roVEMP: repetitive ocular vestibular–evoked myogenic potentials
SFEMG: single-fiber EMG
STARD: Standards for Reporting of Diagnostic Accuracy Studies

## References

[1] Valko Y, Rosengren SM, Jung HH, Straumann D, Landau K, Weber KP. Ocular vestibular evoked myogenic potentials as a test for myasthenia gravis. Neurology. 2016;86(7):660-668. doi:10.1212/WNL.0000000000002383 26791146

[2] Wirth MA, Valko Y, Rosengren SM, et al. Repetitive ocular vestibular evoked myogenic potential stimulation for the diagnosis of myasthenia gravis: optimization of stimulation parameters. Clin Neurophysiol. 2019;130(7):1125-1134. doi:10.1016/j.clinph.2019.03.033 31082787

[3] De Meel RHP, Keene KR, Wirth MA, et al. Repetitive ocular vestibular evoked myogenic potentials in myasthenia gravis. Neurology. 2020;94(16):e1693-e1701. doi:10.1212/WNL.0000000000009306 32217778

[4] Padua L, Stålberg E, LoMonaco M, Evoli A, Batocchi A, Tonali P. SFEMG in ocular myasthenia gravis diagnosis. Clin Neurophysiol. 2000;111(7):1203-1207. doi:10.1016/s1388-2457(00)00307-2 10880794

[5] Whiting PF, Rutjes AW, Westwood ME, et al; QUADAS-2 Group. QUADAS-2: a revised tool for the quality assessment of diagnostic accuracy studies. Ann Intern Med. 2011;155(8):529-536. doi:10.7326/0003-4819-155-8-201110180-00009 22007046

[6] Besinger UA, Toyka KV, Hömberg M, Heininger K, Hohlfeld R, Fateh-Moghadam A. Myasthenia gravis: long-term correlation of binding and bungarotoxin blocking antibodies against acetylcholine receptors with changes in disease severity. Neurology. 1983;33(10):1316-1321. doi:10.1212/wnl.33.10.1316 6684226

[7] Simpson JA. Myasthenia gravis. 1991. Accessed April 15, 2024. hdl.handle.net/1842/27395.

[8] Dias L, Araújo R. Ice pack test in myasthenia gravis: a cool investigation at the bedside. Lancet. 2020;396(10261):e82. doi:10.1016/S0140-6736(20)32263-7 33160580

[9] Pascuzzi RM. The edrophonium test. Semin Neurol. 2003;23(1):83-88. doi:10.1055/s-2003-40755 12870109

[10] AAEM Quality Assurance Committee American Association of Electrodiagnostic Medicine. Literature review of the usefulness of repetitive nerve stimulation and single fiber EMG in the electrodiagnostic evaluation of patients with suspected myasthenia gravis or Lambert-Eaton myasthenic syndrome. Muscle Nerve. 2001;24(9):1239-1247. doi:10.1002/mus.1140 11494281

[11] Jaretzki A III, Barohn RJ, Ernstoff RM, et al. Myasthenia gravis: recommendations for clinical research standards. Task Force of the Medical Scientific Advisory Board of the Myasthenia Gravis Foundation of America. Neurology. 2000;55(1):16-23. doi:10.1212/wnl.55.1.16 10891897

[12] Rosengren SM, Colebatch JG, Straumann D, Weber KP. Why do oVEMPs become larger when you look up? Explaining the effect of gaze elevation on the ocular vestibular evoked myogenic potential. Clin Neurophysiol. 2013;124(4):785-791. doi:10.1016/j.clinph.2012.10.012 23177454

[13] Weber KP, Rosengren SM. Clinical utility of ocular vestibular-evoked myogenic potentials (oVEMPs). Curr Neurol Neurosci Rep. 2015;15(5):22. doi:10.1007/s11910-015-0548-y 25773001

[14] Chihara Y, Iwasaki S, Fujimoto C, Ushio M, Yamasoba T, Murofushi T. Frequency tuning properties of ocular vestibular evoked myogenic potentials. Neuroreport. 2009;20(16):1491-1495. doi:10.1097/WNR.0b013e3283329b4a 19809372

[15] Rosengren SM, Welgampola MS, Colebatch JG. Vestibular evoked myogenic potentials: past, present and future. Clin Neurophysiol. 2010;121(5):636-651. doi:10.1016/j.clinph.2009.10.016 20080441

[16] Stålberg E, Sanders DB, Ali S, et al. Reference values for jitter recorded by concentric needle electrodes in healthy controls: a multicenter study. Muscle Nerve. 2016;53(3):351-362. doi:10.1002/mus.24750 26112058

[17] Leys C, Ley C, Klein O, Bernard P, Licata L. Detecting outliers: do not use standard deviation around the mean, use absolute deviation around the median. J Exp Soc Psychol. 2013;49(4):764-766. doi:10.1016/j.jesp.2013.03.013

[18] Bossuyt PM, Reitsma JB, Bruns DE, et al; Standards for Reporting of Diagnostic Accuracy. Towards complete and accurate reporting of studies of diagnostic accuracy: the STARD initiative. BMJ. 2003;326(7379):41-44. doi:10.1136/bmj.326.7379.41 12511463 PMC1124931

[19] Deeks JJ, Wisniewski S, Davenport C. Chapter 4: guide to the contents of a Cochrane diagnostic test accuracy protocol. In: Deeks JJ, Bossuyt PM, Gatsonis C, eds. Cochrane Handbook for Systematic Reviews of Diagnostic Test Accuracy. The Cochrane Collaboration; 2013.

[20] Hanley JA, McNeil BJ. The meaning and use of the area under a receiver operating characteristic (ROC) curve. Radiology. 1982;143(1):29-36. doi:10.1148/radiology.143.1.7063747 7063747

[21] Obuchowski NA. Sample size calculations in studies of test accuracy. Stat Methods Med Res. 1998;7(4):371-392. doi:10.1177/096228029800700405 9871953

[22] Pewsner D, Battaglia M, Minder C, Marx A, Bucher HC, Egger M. Ruling a diagnosis in or out with “SpPIn” and “SnNOut”: a note of caution. BMJ. 2004;329(7459):209-213. doi:10.1136/bmj.329.7459.209 15271832 PMC487735

[23] Hatanaka Y, Higashihara M, Chiba T, Miyaji Y, Kawamura Y, Sonoo M. Utility of repetitive nerve stimulation test for ALS diagnosis. Clin Neurophysiol. 2017;128(5):823-829. doi:10.1016/j.clinph.2017.02.021 28340431

[24] Abraham A, Alabdali M, Alsulaiman A, et al. Repetitive nerve stimulation cutoff values for the diagnosis of myasthenia gravis. Muscle Nerve. 2017;55(2):166-170. doi:10.1002/mus.25214 27287989

[25] Abraham A, Breiner A, Barnett C, et al. Electrophysiological testing is correlated with myasthenia gravis severity. Muscle Nerve. 2017;56(3):445-448. doi:10.1002/mus.25539 28029691

[26] Lamb CJ, Rubin DI. Sensitivity and specificity of repetitive nerve stimulation with lower cutoffs for abnormal decrement in myasthenia gravis. Muscle Nerve. 2020;62(3):381-385. doi:10.1002/mus.26999 32530515

[27] Yoganathan K, Stevenson A, Tahir A, Sadler R, Radunovic A, Malek N. Bedside and laboratory diagnostic testing in myasthenia. J Neurol. 2022;269(6):3372-3384. doi:10.1007/s00415-022-10986-3 35142871 PMC9119875

[28] Stålberg E, Trontelj JV, Sanders DB. Single Fiber EMG, 3rd ed. Edshagen Publishing House; 2010.

[29] Trontelj JV, Khuraibet A, Mihelin M. The jitter in stimulated orbicularis oculi muscle: technique and normal values. J Neurol Neurosurg Psychiatry. 1988;51(6):814-819. doi:10.1136/jnnp.51.6.814 2841429 PMC1033153

[30] Bruenech JR, Kjellevold Haugen IB. How does the structure of extraocular muscles and their nerves affect their function? Eye (Lond). 2015;29(2):177-183. doi:10.1038/eye.2014.269 25397785 PMC4330282

[31] Horn AK, Leigh RJ. The anatomy and physiology of the ocular motor system. Handb Clin Neurol. 2011;102:21-69. doi:10.1016/B978-0-444-52903-9.00008-X 21601062

[32] Weber KP, Rosengren SM, Michels R, Sturm V, Straumann D, Landau K. Single motor unit activity in human extraocular muscles during the vestibulo-ocular reflex. J Physiol. 2012;590(13):3091-3101. doi:10.1113/jphysiol.2011.226225 22526888 PMC3406392

[33] Govender S, Cheng PY, Dennis DL, Colebatch JG. Electrode montage and gaze effects on ocular vestibular evoked myogenic potentials (oVEMPs). Clin Neurophysiol. 2016;127(8):2846-2854. doi:10.1016/j.clinph.2016.05.365 27417061

[34] Makowiec KF, Piker EG, Jacobson GP, Ramadan NM, Roberts RA. Ocular and cervical vestibular evoked myogenic potentials in patients with vestibular migraine. Otol Neurotol. 2018;39(7):e561-e567. doi:10.1097/MAO.0000000000001880 29912833

[35] Shahnaz N, David EA. Normal values for cervical and ocular vestibular-evoked myogenic potentials using EMG scaling: effect of body position and electrode montage. Acta Otolaryngol. 2021;141(5):440-448. doi:10.1080/00016489.2021.1887517 33641604

[36] Sung PH, Cheng PW, Young YH. Effect of gender on ocular vestibular-evoked myogenic potentials via various stimulation modes. Clin Neurophysiol. 2011;122(1):183-187. doi:10.1016/j.clinph.2010.06.004 20591729

